# Atrial Fibrillation Termination as a Predictor for Persistent Atrial Fibrillation Ablation: A Systemic Review and Meta-Analysis of Prospective Studies

**DOI:** 10.1155/2024/9944490

**Published:** 2024-06-21

**Authors:** Jialing He, Zhen Zhang, Duan Luo, Xianchen Yang, Guoshu Yang, Hanxiong Liu

**Affiliations:** ^1^ Department of Cardiology The Affiliated Hospital of Southwest Jiaotong University The Third People's Hospital of Chengdu Cardiovascular Disease Research Institute of Chengdu, Chengdu, China; ^2^ Physical Examination Department Modern Hospital of Sichuan, Chengdu, China

**Keywords:** atrial fibrillation, catheter ablation, recurrence, termination

## Abstract

**Background:** In this systematic review and meta-analysis, we aimed to validate the predictive role of atrial fibrillation (AF) termination in long-term arrhythmia recurrence.

**Method:** Our search encompassed databases including MEDLINE, EMBASE, PubMed, and the Cochrane Library up to August 1, 2021. Three independent reviewers conducted screening and data extraction. The data included ablation strategy, recurrence mode, AF termination mode, numbers of patients, and recurrence cases in the termination and nontermination groups. The primary endpoint was the recurrence of atrial arrhythmia at long-term follow-up (≥ 12 months).

**Results:** Our analysis included 22 publications, with 11 prospective studies being eligible for further meta-analysis. Among these, 14 studies reported significantly lower rates of arrhythmia recurrence in the AF termination group compared to the nontermination group. Among seven studies involving 1114 patients that examined single procedure outcomes, the pooled estimated effect was RR 0.78 (95% CI 0.68–1.90) with an *I*^2^ value of 57%. Subgroup analysis focusing on termination mode as sinus rhythm yielded a pooled estimated effect of RR 0.74 (95% CI 0.59–0.92) with an *I*^2^ value of 47%. Additionally, analysis of seven studies involving 1433 patients for repeat procedures demonstrated a significant preference for the AF termination group (RR 0.83, 95% CI 0.71–0.97, *I*^2^ = 84%). Subgroup analysis indicated reduced heterogeneity when the termination mode was sinus rhythm (RR 0.68, 95% CI 0.51–0.90, *I*^2^ = 57%).

**Conclusion:** Our study establishes that AF termination serves as an effective predictor for the success of persistent AF ablation procedures. This finding holds potential implications for clinical practice and contributes to our understanding of long-term arrhythmia recurrence in the context of AF termination.

## 1. Introduction

Atrial fibrillation (AF) is the most common sustained cardiac dysrhythmia [1]. AF is associated with decreased quality of life, a fivefold increased risk of stroke, increased risk of heart failure and dementia, and mortality [2]. Persistent AF, taking approximately 50% of AF, is associated with higher AF burden and higher stroke and mortality risk compared to paroxysmal AF [3]. However, the aggregate evidence for the efficacy of catheter ablation for persistent AF is weaker [4]. It was reported that 18 months after persistent AF radiofrequency ablation, the recurrence rate of AF of different kind of strategies was about 50% [5], even in the most experienced centers, requiring a mean of 1.8 procedures/patient [6].

People tried to seek for more protective characteristics of persistent AF patients and ablation procedures to avoid atrial arrhythmia recurrence. AF termination as an effective predictor even as an endpoint for persistent AF ablation was reported in previous literatures [6, 7], but the long-term effect on SR maintenance was questioned by some others [8, 9]. As AF termination was hard to achieve during a single procedure, reported AF termination rate ranging from 33% to 86% [10, 11] and coasting longer operation time [8], whether it is worth to acquire AF termination as an endpoint in operation, was doubted. However, once a patient recovers to normal rhythm, the long-term outcome might be better.

We systemically reviewed related documents and implemented a meta-analysis on prospective studies, trying to identify the effect of AF termination as a predictor on long-term arrhythmia recurrence.

## 2. Method

### 2.1. Study Selection

We searched PubMed, MEDLINE, EMBASE, and Cochrane databases (inception to August 1, 2021) using the terms “atrial fibrillation,” “ablation” OR “catheter ablation,” and “termination.” In addition, we reviewed the reference lists of retrieved studies and major conference proceedings. Any article that met the criteria listed in the following section was retrieved. When groups published multiple reports with overlapping cohorts, the most recent study was included. The inclusion criteria were as follows:

1. Study population should be persistent AF.
2. Endpoints must be AF/atrial arrhythmia recurrence or freedom from atrial arrhythmia or sinus maintenance.
3. Arrhythmia recurrence was defined as any episode of documented AF or atrial tachycardia (AT) or atrial flutter (AFL) > 30 s after the initial 3-month blanking period.
4. Termination of AF should be achieved by ablation or ablation plus electronic cardioversion at the end of ablation, instead of electronic cardioversion only.
5. AF termination group must consist of patients with AF termination during operation entirely.
6. Specific numbers of recurrence were recorded, respectively, in AF termination and AF nontermination groups.
7. Follow-up duration should be over 12 months.
8. Types of study should be an observational study or clinical trial.
9. Full text of study should be available.
10. English publication.

Quality assessment was accomplished with the use of the Newcastle–Ottawa scale for nonrandomized studies by three reviewers (JL H, D L, and GS Y). Agreement between all three reviewers was mandatory for the final classification of the studies. The data used for the analysis are specifically referenced within the work and freely available to all researchers.

### 2.2. Data Extraction

Three authors (JL H, D L, and GS Y) performed database searches independently with agreement on the inclusion of the selected studies. Data extraction and preparation of this article followed the recommendations of the PRISMA group [12]. Data on study type, procedure strategies, termination mode, recurrence mode, follow-up duration, cardioversion implementation, termination and nontermination group population, and outcomes were entered independently by three authors and reviewed for discrepancies. In studies permitting repeat ablations, outcomes for both of the first and repeat ablations were extracted.

### 2.3. Statistical Analysis

Continuous variables were presented as mean ± SD. Dichotomous values were expressed as *n* (%). The Chi-square test was used to compare categorical variables. Analyses were performed using Reviewer Manager, version 5.4. The Mantel–Haenszel method for dichotomous data was used to calculate the 95% confidence interval (CI) and aggregated risk ratio (RR). Statistical heterogeneity on each outcome of interest was quantified using the *P* value for the *Q* statistic and *I*^2^. Heterogeneity based on *I*^2^ was considered low if < 25%, moderate if 25% to 75%, and high if > 75%. Summary estimates were presented as forest plot and performed a fixed (*I*^2^ < 50%) or a random(*I*^2^ > 50%) effects model. If substantial between-study heterogeneity was observed, subgroup analysis was undertaken to identify the source of heterogeneity. Stepwise exclusion of 1 study at a time was used to perform a sensitivity analysis to determine the impact of an individual study on the outcomes.

## 3. Results

**Figure 1:**
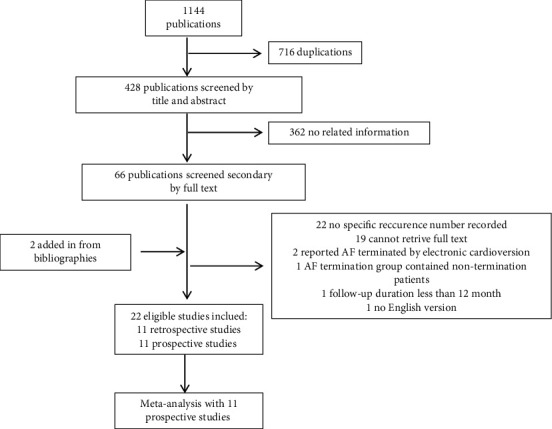
Flow chart of study selection.

A total of 1144 publications were searched including 716 duplications. The rest 428 articles were included in primary selection through title and abstract, and 360 articles were expelled because of no related information reported. We secondary reviewed 68 publications and excluded 46 (22 do not report specific recurrence numbers of AF patients, 19 cannot retrieve full text, 2 reported AF terminated by electronic cardioversion only, 1 without long-term outcome, 1 not English version, and 1 with overlapping population). Another 2 studies were identified from a review of bibliographies. The flow chart of study inclusion and exclusion is shown in Figure 1.

### 3.1. Study Characteristics

We finally included 22 articles [6, 10, 11, 13–31]. Eleven studies were prospective which would be further applied for meta-analysis. The study quality is shown in Table S1. The characteristics of each study are listed in Table 1. Most of the studies took stepwise ablation protocol. There was a divergence on the AF termination mode, which was sinus rhythm (SR) directly or SR/AT.

In most of the studies, AF was terminated by ablation, and only four studies achieved termination by ablation plus subsequent cardioversion. Fourteen articles reported single procedure outcome including seven prospective studies, and 11 articles including seven prospective studies record repeat procedure outcome.

The follow-up duration differed among those articles, but the majority of studies had a median follow-up duration greater than 12 months. Only 1.9% of patients developed arrhythmia again reported by Haissaguerre et al. with a median 11 ± 6-month follow-up after repeat procedures [13]. And the highest recurrence rate was seen in the study of Scherr et al., at the 5^th^ year after AF ablation with 87.5% recurrence patients in the termination group [19].

There were 14 researches reported a significantly lower arrhythmia recurrence rate in the AF termination group than in the nontermination group, including six prospective studies. Matsuo et al. reported that only three out of 76 AF termination patients had recurrence, while 13 out of 14 nontermination patients got arrhythmia during a mean 28 ± 4-month follow-up (3.9% vs 92.3%, *P* < 0.00001) [23]. Although the difference of arrhythmia recurrence between the AF termination and nontermination groups was not significant in the rest of the eight studies, the absolute recurrence rate was lower in the AF termination group, meaning slightly but not obviously more patients were arrhythmia-free if AF termination was achieved in operation. Singh et al. (51.4% vs 50.5%, *P* = 0.9) [21] and Choi et al. (3-year follow-up 73.2% vs 72.5% *P* = 0.89) reported that slightly more patients got recurrence in the AF termination group [30].

Choi et al., Miyazaki et al., and Scherr et al. reported a long-term outcome over 4 years and displayed it annually [19, 29, 30]. According to Miyazaki et al.(1-year follow-up 11.6% vs 18.2% *P* = 0.3; 2-year follow-up 20.3% vs 37.9% *P* = 0.02; 3-year follow-up 24.6% vs 54.5% *P* = 0.0005; 5-year follow-up 28.9% vs 68.2% *P* < 0.00001) [29] and Scherr et al. (1-year follow-up 61.7% vs 76.7% *P* = 0.07; 2-year follow-up 68.3% vs 87.7% *P* = 0.01; 3-year follow-up 24.6% vs 54.5% *P* = 0.0005; 5-year follow-up 28.9% vs 68.2% *P* < 0.00001) [19], a significant superiority on arrhythmia recurrence of the AF termination group over the nontermination group started to appear at the 2nd year after ablation. However, in Choi et al.'s study [30], the difference did not appear during the whole 5-year follow-up.

### 3.2. Meta-Analysis of Prospective Studies

**Figure 2:**
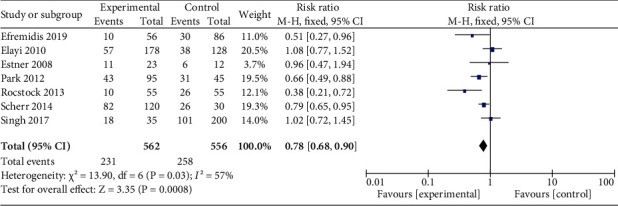
Forest plot for single procedure outcome. Result of the effect of AF termination on recurrence for the single ablation procedure.

There were seven studies that recorded single procedure outcome [14–16, 18, 19, 21, 22], involving 1114 patients. Because the median follow-up time for the remaining studies was around 20 months, as for the study of Scherr et al., we included data from the 2nd year to perform meta-analysis. The final pooled estimated effect was RR 0.78【95% CI 0.68–0.90】 with *I*^2^ = 57% (Figure 2). Subgroup analysis was based on termination mode, recurrence type, and ablation strategy (Table 2). The pooled estimated effect was RR 0.74【95% CI 0.59–0.92】 with *I*^2^ = 47% in the subgroup of termination mode as SR. We further conducted a sensitive analysis, and the results remained stable through omitting study one by one.

**Figure 3:**
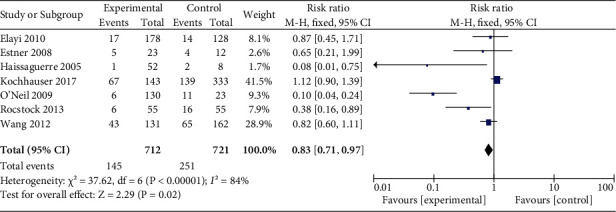
Forest plot for repeat procedure outcome. Result of the effect of AF termination on recurrence for repeat ablation procedure.

As for the effect of AF termination on repeat ablation outcome, seven studies comprising 1433 patients were included [6, 13–15, 17, 18, 20]. The pooled RR was 0.83 【95% CI 0.71-0.97】 with high heterogeneity (*I*^2^ = 84%) (Figure 3). The results and heterogeneity did not change significantly during sensitive analysis.

Next, we conduct the subgroup analysis (Table 3). SR mode subgroup demonstrated lower heterogeneity (*I*^2^ = 57%) with a pooled estimated effect of RR 0.68 (95% CI 0.51–0.90).

## 4. Discussion

We reviewed studies comparing long-term recurrence of AF termination and nontermination patients during ablation, noticing that most researches indicated AF termination patients encountering significantly less atrial arrhythmia recurrence. In the studies that showed a nonsignificant difference between the AF termination and nontermination groups, interestingly, we notice that the absolute number of patients with recurrence in the AF termination group always seemed to be less than in the AF nontermination group. In the further meta-analysis with prospective studies, we found that the long-term prognosis of patients in the AF termination group was significantly better than that in the nontermination group.

Several studies also reported AF termination as a multivariant independent predictor for arrhythmia-free survival [16, 18, 19, 29, 32–34]. However, termination as an endpoint was under doubt. Wang et al. randomized 400 patients to the technical endpoint group and pursuing AF termination group, and freedom from atrial arrhythmias did not differ between the two groups after a single ablation procedure (46.5% vs. 54.3%, *P* = 0.12) and the final ablation procedure (60.1% vs. 65.8%, *P* = 0.24) [8]. But in Wang et al.'s study, only 63.1% of the AF termination group patients achieved SR or AT versus 27.2% of that in the control group, which means that the comparison of AF termination and AF nontermination was indirect and could not explain the real effect of AF termination. Li et al. conducted a meta-analysis of RCT and concluded that AF termination was not a reliable procedural endpoint during ablation of persistent AF [9]. However, we found that the measures terminating AF defined by Li et al. were not only ablation, but also purely electrical cardioversion. We further read these articles containing data on electrical cardioversion and found that if ablation terminating AF could not be completed, electrical cardioversion alone did not appear to improve outcomes [35, 36].

The mode of AF termination also matters. Our meta-analysis implied that SR as a termination mode was associated to significantly less recurrence during long-term follow-up both in single and repeat procedures. Estner et al. showed after 19 ± 12-month follow-up that 83.5% of patients achieving SR through ablation were free from atrial arrhythmia recurrence, while only 73.3% of patients with AT during ablation were free from atrial arrhythmia recurrence [14]. Efremidis et al. reported 64.9% versus 77.8% freedom from recurrence after 21.1 ± 0.8 months, respectively, in SR and AT mode [22]. Ammar et al. found that AF termination into SR was associated with a lower risk of arrhythmia recurrence even after adjustment for age, sex, diabetes mellitus, arterial hypertension, LA diameter, and AF duration (hazard ratio, 0.62; *P* = 0.04) [11].

The mechanisms of AF are recognized as one part responsible for its initiation and another part responsible for its perpetuation. In the early stages, it triggers promoted electrical activity and initiated paroxysmal AF. The ectopic triggers were usually the pulmonary vein and can also be the vena cava, the crista terminalis, the coronary sinus, the ligament of Marshall, the interatrial septum, and the appendages. Once induced, AF may be self-perpetuating by several mechanisms, which was under debate. Multiple wavelets and localized focal or reentrant sources are largely accepted to drive AF [37].

Theoretically, the elimination of the perpetuated substrate can directly lead to the termination of persistent AF. And once the driver area was dispelled, recurrence would seldom happen. According to Haissaguerre et al., termination of AF was predictive of noninducibility after ablation and was associated with improved long-term outcomes (87% versus 63% freedom from recurrent AF; *P* = 0.03) [38]. So the key point was how to define the perpetuated substrate. One promising method was to identify the myocardial fibrosis area. Fibrotic atrial structural remodeling was verified by histological examination and autopsy in AF patients [39]. Fibrosis was considered to perpetuate AF through mechanisms including acting as boundaries critical to anchor rotors, facilitating transient reentrant circuits, and promoting rapid repetitive activity through microreentry or local automaticity [40]. Low voltage zones (LVZ) have previously shown to be correlated with areas presenting delayed enhancement on magnetic resonance imaging scar [41]. The efficacy of voltage-guided substrate modification by targeting LVZ was controversial [42, 43]. We still cannot neglect the fact that the definition of LVZ was obscure, as no clear cut-off values for significant fibrosis were accepted. Most studies use a 0.5 mV cut-off empirically, without any histological evidence [41, 44]. Our center found a new measurement to define LVZ through a kind of individualized index. We implemented a single-center cohort study with 280 patients, which will be publicated soon, and results showed that 90% of patients achieved acute AF termination into SR and 82% of patients were in long-term success.

Our study is one of the few meta-analyses focused on the AF termination and ablation long-term prognosis. We found that patients with AF termination had lower long-term recurrences. At the same time, we found new revelations that the mode of AF termination was important, and SR may be more likely to maintain arrhythmia-free survival. Our study recognized that AF termination could be an indicator for ablation outcome.

The main limitations of our study were as follows. First, it lacked of researches in the recent 5 years. Second, the high heterogeneity cannot be well explained. Third, this study was a study-level meta-analysis rather than a patient-level meta-analysis which reduced study reliability.

## 5. Conclusion

Our study found that AF termination was an effective predictor for persistent AF patients with significantly less recurrence in long-term follow-up regardless of single or repeat procedure.

## Figures and Tables

**Table 1:** Characteristics of all studies.

**Name/year**	**Study type**	**Ablation strategy**	**ADD application after ablation**	**Recurrence mode**	**Cardioversion in termination group**	**Termination mode**	**Follow-up (m)**	**Single procedure**					**Repeat procedure**				
								**Termination group ** ***n*** **(%)**	**Termination group recurrence ** ***n*** **(%)**	**Nontermination group ** ***n*** **(%)**	**Nontermination group recurrence ** ***n*** **(%)**	**P** **value**	**Termination group ** ***n*** **(%)**	**Termination group recurrence ** ***n*** **(%)**	**Nontermination group ** ***n*** **(%)**	**Nontermination group recurrence ** ***n*** **(%)**	**P** **value**
Haissaguerre 2005 [13]	Prospective	Stepwise	2 months	AF/AT/AFL	No	SR	11 ± 6	—	—	—	—	—	52	1 (1.9%)	8	2 (25%)	< 0.0000
Estner 2008 [14]	Prospective	PVI + CFAE	Yes	AF/AT/AFL	No	SR、AT	19 ± 12	23	11 (47.8%)	12	6 (50%)	0.9	23	5 (21.8%)	12	4 (33.3%)	0.45
						SR		8	4 (50%)	12	6 (50%)	1	8	1 (12.5%)	12	4 (33.3%)	0.34
O'Neil 2009 [6]	Prospective	Stepwise	Yes	AF/AT/AFL	Yes (15%)	SR, AT	30 ± 11	—	—	—	—	—	130	6 (4.6%)	23	11 (47.8)	< 0.0000
Elayi 2010 [15]	Prospective	PVI + CFAE	Yes	AF/AT/AFL	No	SR, AT	25 ± 6.9	178	57 (32%)	128	38 (29.7%)	0.66	178	17 (10%)	128	14 (11%)	0.69
Wang 2012 [17]	Prospective	Stepwise	NA	AF/AT/AFL	No	SR	23 ± 7	—	—	—	—	—	131	43 (32.8%)	162	65 (40.1%)	0.20
Park 2012 [16]	Prospective	Stepwise	3 months	AF/AT/AFL	No	SR	18.7 ± 7.6	95	43 (45.3%)	45	31 (68.9%)	0.005					
Rostock 2013 [18]	Prospective	Stepwise	3 months	AF	No	SR	20.1 ± 13.3	55	10 (18.2%)	55	26 (47.3%)	0.002	55	6 (10.9%)	55	16 (29.9%)	0.02
Scherr 2014 [19]	Prospective	Stepwise	No	AF/AT/AFL	No	SR	12	120	74 (61.7%)	30	23 (76.7%)	0.07	—	—	—	—	—
							24	120	82 (68.3%)	30	26 (87.7%)	0.01	—	—	—	—	—
							26	120	90 (75%)	30	27 (90%)	0.02	—	—	—	—	—
							48	120	95 (79.2%)	30	29 (96.7%)	0.0006	—	—	—	—	—
							60	120	105 (87.5%)	30	29 (96.7%)	0.001	—	—	—	—	—
Kochhauser 2017 [20]	Prospective	PVI/PVI + CFAE/PVI + LL	3 months	AF	Yes	SR, AT	18	—	—	—	—	—	143	67 (46.9%)	333	193 (57.9%)	0.5
Singh 2017 [21]	Prospective	PVI	No	AF/AT/AFL	No	SR, AT	12	35	18 (51.4%)	200	101 (50.5%)	0.9	—	—	—	—	—
Efremidis 2019 [22]	Prospective	Stepwise	3 months	AF/AT/AFL	No	SR, AT	21.1 ± 0.8	56	10 (17.9%)	84	30 (35.7%)	0.03	—	—	—	—	—
						SR		36	8 (22.2%)	84	30 (35.7%)	0.16	—	—	—	—	—
Matsuo 2009 [23]	Retrospective	Stepwise	No	AF	No	SR	28 ± 4	76	3 (3.9%)	14	13 (92.3%)	< 0.0000	—	—	—	—	—
Hocini 2010 [10]	NA	Stepwise	No	AF/AT/AFL	No	SR, AT	22 ± 9	128	67 (52.3%)	20	18 (90%)	< 0.0000	—	—	—	—	—
Heist 2012 [24]	Retrospective	Stepwise	2 months	AF/AT/AFL	No	SR, AT	2 to 33	—	—	—	—	—	59	46 (77.9%)	48	28 (58.3%)	0.03
Komatsu 2012 [25]	Retrospective	Stepwise	No	AF/AT/AFL	Yes (21.6%)	SR, AT	20 ± 11	51	25 (49%)	81	68 (83.9%)	< 0.0000	—	—	—	—	—
Ammar 2013 [11]	NA	PVI + CFAE+LL	No	AF/AT/AFL	No	SR	12	62	26 (41.9%)	129	26 (20.2%)	0.002	—	—	—	—	—
Zhou 2013 [26]	Retrospective	Stepwise	NA	AF/AT/AFL	No	SR	50 ± 9.3	94	30 (31.9%)	106	67 (63.2%)	0.04	—	—	—	—	—
Kumagai 2013 [31]	Retrospective	Stepwise	3 months	AF	No	NA	12 ± 4						14	4 (28.6%)	56	32 (57.1%)	0.06
Pascale 2014 [27]	NA	Stepwise	3 months	AF/AT/AFL	No	SR, AT	36 ± 8	73	13 (17.8%)	24	12 (50%)	0.003	73	14 (19.2%)	24	15 (62.5%)	0.0003
Wu 2014 [28]	Retrospective	Linear	No	AF/AT/AFL	No	SR	5.1 year	75	37 (49.3%) No	45	11 (24.4%)	0.008	—	—	—	—	—
Miyazaki 2017 [29]	NA	PVI + substrate modification	3 months	AF/AT/AFL	Yes	SR + AT	12	69	8 (11.6%)	66	12 (18.2%)	0.3	—	—	—	—	—
							24	69	14 (20.3%)	66	25 (37.9%)	0.02	—	—	—	—	—
							36	69	17(24.6%)	66	36 (54.5%)	0.0005	—	—	—	—	—
							60	69	20 (28, 9%)	66	45 (68.2%)	<0.0000	—	—	—	—	—
Choi 2019 [30]	Retrospective	PVI + substrate modification+ablation of inferior line, CFAE, and common origin sites	Yes	AF/AT/AFL	No	SR	12	112	37 (33%)	138	56 (40.6%)	0.22	—	—	—	—	—
							24	112	63 (56.3%)	138	80 (57.9%)	0.78	—	—	—	—	—
							36	112	82 (73.2%)	138	100 (72.5%)	0.89	—	—	—	—	—
							48	112	96 (85.7%)	138	116 (84.1%)	0.72	—	—	—	—	—

**Table 2:** Results of subgroup analysis for a single procedure.

**Subgroup**	**RR**	**95% CI**	**I** ^2^
Termination			
AT+SR	0.68	0.68–0.90	57%
SR	0.74	0.59–0.92	47%
Recurrence			
Atrial arrhythmia	0.79	0.64–0.98	50%
Atrial fibrillation	0.38	0.21–0.72	—
Ablation strategy			
Stepwise	0.62	0.52–0.74	67%
Others	0.96	0.70–1.32	35%

**Table 3:** Results of subgroup analysis for repeat procedure.

**Subgroup**	**RR**	**95% CI**	**I** ^2^
Termination			
AT+SR	0.91	0.75–1.11	89%
SR	0.68	0.51–0.90	57%
Recurrence			
Atrial arrhythmia	0.66	0.52–0.85	83%
Atrial fibrillation	0.81	0.77–0.97	84%
Ablation strategy			
Stepwise	0.58	0.44–0.75	84%
Others	1.08	0.88–1.32	0%

## Data Availability

Data is available on request from the authors.

## Supporting Information

Supporting Information Additional supporting information can be found online in the Supporting Information section. Table S1 Quality assessment table: table of quality assessment of included studies by the Newcastle–Ottawa scale.
