# Supplementary material for: Atrial Fibrillation Termination as a Predictor for Persistent Atrial Fibrillation Ablation: A Systemic Review and Meta-Analysis of Prospective Studies
Source: Cardiovasc Ther. 2024 Jun 21;2024:9944490. doi: 10.1155/2024/9944490 (PMC11213638; doi:10.1155/2024/9944490)
Supplement: Supporting Information — Additional supporting information can be found online in the Supporting Information section. Table S1 Quality assessment table: table of quality assessment of included studies by the Newcastle–Ottawa scale. [file 9944490.f1.doc]

| Study | Selection | | | | Comparability | Exposure | | | All score |
| --- | --- | --- | --- | --- | --- | --- | --- | --- | --- |
| Haissaguerre2005 | a^#^ | b^#^ | c^#^ | d^#^ | e^#^ | f^#^ | g^#^ | h^#^ | 8* |
|  | * | * | * | * | * | * | * | * |  |
| Estner2008 | * | * | * | * | * | * | * | * | 8* |
| O’Neil2009 | * | * | * | * | ** | * | * | * | 9* |
| Elayi 2010 | * | * | * | * | * |  | * | * | 7* |
| Park2012 | * | * | * | * | ** |  | * | * | 8* |
| Wang2012 | * | * | * | * | ** |  | * | * | 8* |
| Rocstock2013 | * | * | * | * | ** | * | * | * | 9* |
| Scherr2014 | * | * | * | * | ** | * | * | * | 9* |
| Kochhauser  2017 | * | * | * | * | * | * | * | * | 8* |
| Singh2017 | * | * | * | * | ** | * | * | * | 9* |
| Efremidis2019 | * | * | * | * | * | * | * | * | 8* |

*：stand for the study can be awarded one star in corresponding question; a^#^: case definition ; b^#^:Representative of the case; c^#^:Selection of control; d^#^:Definition of control; e^#^:Study control age and sex to reduce bias; Study control other complication as additional factors; f^#^:Ascertainment of exposure; g^#^:Same method of ascertainment for cases and control; h^#^:Non-respond rate
